# Secreted Factors from Bone Marrow Stromal Cells Upregulate IL-10 and Reverse Acute Kidney Injury

**DOI:** 10.1155/2012/392050

**Published:** 2012-12-19

**Authors:** Jack M. Milwid, Takaharu Ichimura, Matthew Li, Yunxin Jiao, Jungwoo Lee, Joshua S. Yarmush, Biju Parekkadan, Arno W. Tilles, Joseph V. Bonventre, Martin L. Yarmush

**Affiliations:** ^1^Center for Engineering in Medicine and Surgical Services, Massachusetts General Hospital, Harvard Medical School, 51 Blossom Street, Boston, MA 02114, USA; ^2^Harvard-MIT Division of Health Sciences and Technology, Massachusetts Institute of Technology, Cambridge, MA 02139, USA; ^3^Renal Medicine Division, Brigham and Women's Hospital, Harvard Medical School, Boston, MA 02115, USA; ^4^Department of Research and Development, Sentien Biotechnologies, Inc., Medford, MA 02155, USA; ^5^Department of Biomedical Engineering, Rutgers University, Piscataway, NJ 08901, USA

## Abstract

Acute kidney injury is a devastating syndrome that afflicts over 2,000,000 people in the US per year, with an associated mortality of greater than 70% in severe cases. Unfortunately, standard-of-care treatments are not sufficient for modifying the course of disease. Many groups have explored the use of bone marrow stromal cells (BMSCs) for the treatment of AKI because BMSCs have been shown to possess unique anti-inflammatory, cytoprotective, and regenerative properties *in vitro *and *in vivo*. It is yet unresolved whether the primary mechanisms controlling BMSC therapy in AKI depend on direct cell infusion, or whether BMSC-secreted factors alone are sufficient for mitigating the injury. Here we show that BMSC-secreted factors are capable of providing a survival benefit to rats subjected to cisplatin-induced AKI. We observed that when BMSC-conditioned medium (BMSC-CM) is administered intravenously, it prevents tubular apoptosis and necrosis and ameliorates AKI. In addition, we observed that BMSC-CM causes IL-10 upregulation in treated animals, which is important to animal survival and protection of the kidney. In all, these results demonstrate that BMSC-secreted factors are capable of providing support without cell transplantation, and the IL-10 increase seen in BMSC-CM-treated animals correlates with attenuation of severe AKI.

## 1. Introduction

Each year approximately 2,000,000 people in the US develop acute kidney injury (AKI), with a likelihood of death greater than 70% in cases of dialysis dependence [1, 2]. AKI is an inflammatory organ failure syndrome that is caused by extensive injury to the kidney parenchyma [3]. There are various etiologies of AKI that stem from prerenal, intrinsic renal, or postrenal causes. Regardless of etiology, however, it is thought that there is persistent systemic inflammation that may contribute to prevention of tissue regeneration and disease reversal [4]. Resolution may be achievable if the inflammatory cytokine storm created by AKI can be attenuated, and support is provided to aid in the healing of the injured tissue [5]. Unfortunately, the only existing therapies available to patients with AKI are palliative. Dialysis, while useful to restore electrolyte balance and remove waste products in the blood, weakly supplements the failing kidneys without providing anti-inflammatory or regenerative support [6]. Single molecule therapies directed against AKI have thus far proven insufficient for addressing the complex pathophysiology underlying the disease [7]. New therapies that can resolve the disease by addressing multiple aspects of AKI pathophysiology in parallel are desperately needed.

Bone marrow stromal cells (BMSCs) have been explored in preclinical and clinical studies as a potential therapeutic for patients with AKI [8]. BMSCs have been shown to possess potent immunomodulatory and regenerative properties *in vitro* and *in vivo* [9, 10]. The predominant mechanisms of BMSC therapy are unclear. Some studies implicate homing, engraftment and differentiation as critical for therapeutic benefit [11], and others stress the importance of secreted factors from BMSCs as the key mediators [12]. To attempt to resolve this issue, we and others have tested BMSC-secreted factors in various disease models and compared them to whole cell transplantation. These studies have demonstrated that BMSC-secreted factors possess the capacity to provide therapeutic support to rodents suffering from acute diseases associated with inflammation (e.g., liver failure, sepsis), and can provide superior support when compared to cell transplantation [13–15]. In particular, we have seen that BMSC-secreted factors are responsible for a reversal of proinflammatory cytokine expression and a concomitant increase in the anti-inflammatory cytokine interleukin-10 (IL-10). To date, it has not yet been definitively resolved whether this is also the case in AKI. It has been shown in several models of AKI that these factors are capable of providing significant support [16–18], as measured by a reduction in the total injury to the renal tubule epithelium, and preservation of glomerular filtration rate and creatinine clearance in ischemic and rhabdomyolytic injury models. Tögel and colleagues [16] and Duffield et al. [19] demonstrated that when BMSCs are infused in the context of AKI, very few cells engraft within hours of transplantation, and few cells can be detected in the kidneys after 24 hours which is consistent with other studies that also suggest the half life of BMSCs *in vivo* is on the order of days [20, 21]. Nevertheless, they still observed improvement of the structure and function of kidneys during ischemic AKI. In addition, they observed a similar reduction of the proinflammatory injurious state during AKI as we have seen in other inflammatory disease models with BMSC-secreted factors, as evidenced by suppression of proinflammatory cytokine expression and upregulation of the expression of anti-inflammatory mediators in the kidney at the level of mRNA, including IL-10.

In the current paper, we demonstrate that BMSC-secreted factors are capable of protecting rats from developing severe AKI after cisplatin treatment and can provide a survival benefit without cell transplantation. Compared to vehicle and a mock cell control, we saw a statistically significant survival benefit conferred by BMSC conditioned medium (CM), together with a reduciton of both histological and serum kidney function parameters. In addition, we observed a significant increase in serum IL-10 levels in animals treated with BMSC-CM, and when IL-10 was neutralized using an antibody, the effects of the BMSC-CM were significantly abated. The results of this study clearly indicate that in AKI, the secreted factors alone are sufficient for reduction of injury and prolonged survival.

## 2. Methods

### 2.1. Cell Culture and Conditioned Medium

BMSCs were isolated, purified, grown, and characterized, and fibroblasts grown as described previously [22, 23]. Briefly, BMSCs were isolated from whole bone marrow (Lonza, Basel, Switzerland) by selective adhesion and expanded in colonies for seven days. Colonies were trypsinized and replated for further expansion. Purity of culture (>99%) was determined with flow cytometry (results not shown) by staining BMSCs with BD Pharmingen CD44, CD45, CD29, CD73, CD106, or CD11b antibodies (BD, Franklin Lakes, NJ). All BMSCs were used at passage 2–5. Fibroblasts were cultured from the cell line PCS-201-012 (ATCC, Manassas, VA). The conditioned medium was collected and concentrated as described previously [24]. Briefly, both cell types (BMSCs and fibroblasts) were grown to 60% confluence, rinsed with PBS twice, and then incubated for 24 hours in the presence of serum-free DMEM containing 0.05% bovine serum albumin (Sigma, St. Louis, MO). The conditioned medium was collected and concentrated using Amicon centrifugal concentrators with a 3 kDa cutoff (Millipore, Billerica, MA). Other than culturing in the presence of different cells, conditioned media from BMSCs and fibroblasts (FBs) were collected and prepared identically. By convention, “1X” refers to the concentration of conditioned medium achieved when 15 mL of conditioning medium was incubated in the presence of 2 × 10^6^ cells for 24 hours, collected, and concentrated to a final volume of 1 mL. This preparation was used for both BMSCs and fibroblasts for the purposes of these studies.

### 2.2. Rat Model of Cisplatin-Induced Acute Kidney Injury and BMSC-CM Treatment

All animal studies were performed in accordance with the animal rights policies of the Massachusetts General Hospital Subcommittee on Research Animal Care. Male SAS-SD rats (Charles River Laboratories, Wilmington, MA) weighing 275–300 g were given cisplatin (7.5 mg/kg; Sigma, St. Louis, MO) dissolved in physiological saline via i.p. injection to induce AKI. At various time points after injection, BMSC-CM, FB-CM, physiological saline, or mixtures of BMSC-CM plus anti-rat IL-10Ab (BD, Franklin Lakes, NJ; Invitrogen, Carlsbad, CA) were administered intravenously via penile vein as treatments for the cisplatin-induced AKI. All BMSC-CM and FB-CM doses consisted of 1 mL of 1X CM per dose, and in all cases, the concentration of IL-10Ab in each dose was 4 *μ*g diluted in 1 mL of conditioned medium.

### 2.3. Tissue Histology and Staining

Rats were anesthetized with isofluorane (Hospira, Lake Forest, IL), and laparotomy was performed to access the abdominal cavity. Euthanasia was performed by severing the abdominal aorta and puncturing the diaphragm. Both kidneys were excised and rinsed with physiological saline before fixation in buffered formaldehyde (Sigma, St. Louis, MO) for one week at room temperature. The kidneys were then embedded in paraffin wax and 4 *μ*m sections were created using a microtome. The sections were then analyzed by either (1) staining with hematoxylin and eosin (H&E), (2) TUNEL according to the manufacturer's instructions (R&D Systems, Minneapolis, MN), (3) probed with PCNA antibody as described previously [25], or (4) stained for kidney injury molecule-1 (KIM-1) [26]. All tissues were collected from euthanized animals 5 days following i.p. injection of cisplatin. For the H&E sections, histological scoring was performed on rats subjected to AKI and treated with either saline, BMSC-CM or BMSC-CM + anti-rat IL-10 antibodies, three rats per group, respectively. Three microscopic fields at 40X magnification on a Nikon Optiphot-2 were analyzed per animal for each of three regions of the kidneys, outer cortex (OC), inner cortex (IC), and outer stripe of the outer medulla (OSOM). Tubular injury was quantified based on observed dilation, loss of brush border, cast, debris, thinning, and cell loss. The quantitative score was based on the following metric: 0 = normal; 1 = 1–25% injury; 2 = 26–50% injury; 3 = 51–75% injury; 4 = 76–100% injury. Apoptosis was quantified by taking the average number of TUNEL positive cells in four fields at 10X of the medulla and cortex of rats subjected to cisplatin AKI and treated with either saline (*n* = 4) or BMSC-CM (*n* = 3). For the PCNA staining, we used an anti-PCNA clone PC10 (Sigma, St. Louis, MO), followed by a secondary sheep anti-mouse (1 : 50; Chemicon, Temecula, CA), and signal development using the Vectastain ABC kit (Vector Labs, Burlingame, CA). To determine the number of PCNA positive cells per field, representative images were taken at 20X magnification of the kidney samples for each of three animals, 5 images of the medulla and 5 images of the cortex, and the numbers of positive cells per field were counted and tabulated. Kidney injury molecule-1 KIM-1 was stained as described previously [26, 27]. Briefly, 4 *μ*m paraffin sections were rehydrated and KIM-1 was labeled with a goat anti-rat Tim-1/KIM-1 polyclonal antibody (R&D Systems, Minneapolis, MN). The secondary antibody used was donkey anti-goat Biotin-SP-conjugated, and the signal was developed using ABC peroxidase DAB labeling. The sections were then counterstained with hematoxylin. For TUNEL, PCNA, and KIM-1 staining, isotype controls were performed, in addition to a healthy kidney control for comparison (results not shown).

### 2.4. Blood Urea Nitrogen (BUN) and Creatinine Measurement

All BUN and creatinine measurements were conducted with blood drawn from the rats and analyzed on a Renal Function Panel according to manufacturer's instructions (Abaxis, Union City, CA). For BUN and creatinine analysis, all animals were sacrificed five days after i.p. injection of cisplatin and induction of AKI.

#### 2.4.1. IL-10 ELISA

The rat IL-10 ELISA kit used was provided by a commercial vendor and was used according to the manufacturer's instructions (OptEIA ELISA kit; BD, Franklin Lakes, NJ). Blood was drawn from animals via tail snip on day 3 and sera were analyzed for the presence of IL-10 using this commercial kit. BMSC-CM was also analyzed for IL-10 using the same kit to assess whether the measured IL-10 in the sera of the animals was attributed to the BMSC-CM.

#### 2.4.2. Statistics

Unless otherwise noted, all experiments were repeated in quadruplicate, and all data were assessed for significance using a paired, two-tail Student's *t*-test. All graphs display the average value of each experimental group with error bars indicating one standard deviation in the spread of the data.

## 3. Results

### 3.1. BMSC-CM Attenuates Cisplatin-Induced AKI in Rat

In these studies, we chose to use a cisplatin model of AKI in rats. Cisplatin is used routinely in the clinic as a cancer chemotherapeutic agent and remains the standard of care for many malignancies, including ovarian and cervical cancer, because of its strong therapeutic track record [28, 29]. However, one of the most common and dangerous complications of cisplatin administration is nephrotoxicity [30], which can lead to death with AKI. The primary hypothesis tested was whether BMSC-secreted factors, when used to treat lethal cisplatin administration, could provide the multifactorial support required to protect the kidneys and prevent death by organ failure.

**Figure 1:**
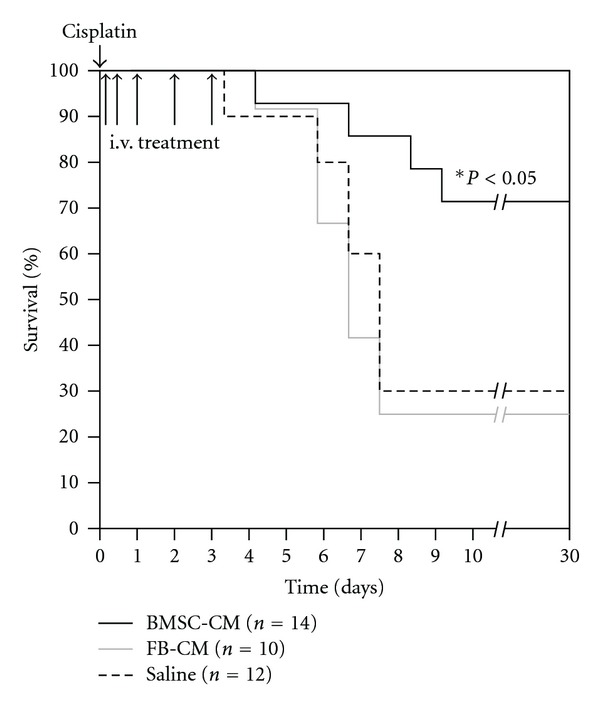
BMSC-CM confers a significant survival benefit to rats undergoing lethal cisplatin*-induced* AKI. At hour 0, cisplatin dissolved in physiological saline (0.75 mg/mL) was administered i.p. to male, SAS-SD rats at a lethal dose (7.5 mg/kg). At hours 3, 9, 24, 48, and 72, 1 mL of either physiological saline (*n* = 12), fibroblast conditioned medium (*n* = 10) or BMSC-conditioned medium (*n* = 14) were administered i.v. to the cisplatin-treated AKI rats. They were monitored for survival for 30 days. *P < 0.05 for BMSC-CM compared to both FB-CM and saline by Logrank test.

**Figure 2:**
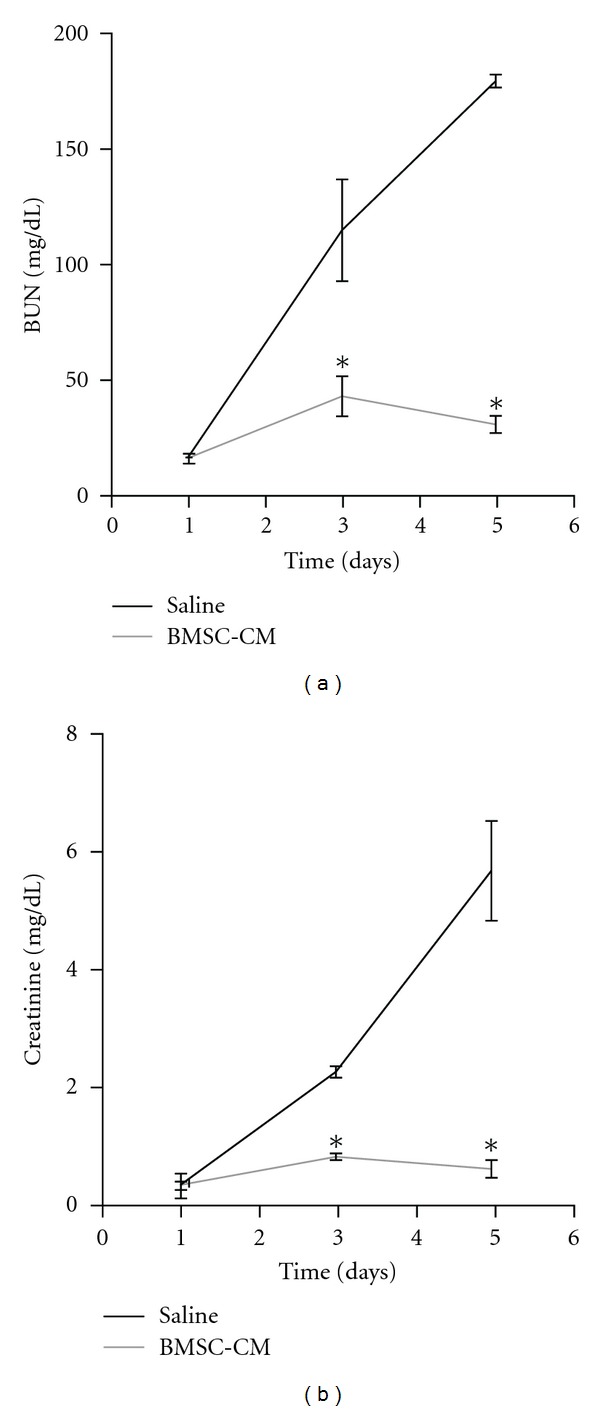
BMSC-CM prevents onset of severe AKI. Blood was collected from rats subjected to lethal cisplatin (7.5 mg/kg) i.p. via tail snip 1, 3, and 5 days following cisplatin administration. The rats were treated at 3, 9, 24, 48, and 72 hours following cisplatin administration, with 1 mL of either physiological saline (*n* = 4), or BMSC-conditioned medium (*n* = 3) i.v. (a) BUN and (b) creatinine were measured to monitor the extent of renal injury as a function of time after cisplatin. *P < 0.01 by Student's *t*-test.

We began our studies by optimizing the cisplatin dose required to induce 75% mortality by two weeks and arrived upon 7.5 mg/kg i.p. [31]. We then determined an effective regimen for treating AKI with BMSC-CM. Previous studies illustrating the kinetics of disease onset demonstrated irreversible damage within the first 72 hours after cisplatin administration [32]. Hence, we chose five time points for repeated i.v. administration of BMSC-CM: 3, 9, 24, 48, and 72 hours after cisplatin was given. This regimen provided a significant survival benefit (Figure 1); compared to controls (FB-CM, *n* = 10; saline, *n* = 12), BMSC-CM (*n* = 14) improved survival from 25% to 71.4% (*P* < 0.05) [22]. In addition to providing a survival benefit, BMSC-CM also significantly attenuated the severity of cisplatin AKI. When animals were administered the same dose of cisplatin, BUN and creatinine rose dramatically in vehicle-treated rats by day 3 (*n* = 4), and continued to climb by day five, indicative of the severity of disease and irreversibility of the injury (Figures 2(a) and 2(b)). However, when the animals were treated with BMSC-CM as described above (*n* = 3), neither BUN nor creatinine rose dramatically at any point during the first five days after cisplatin administration. This indicated that BMSC-CM prevented extensive injury of the kidneys and preserved enough of the architecture and function of the kidneys such that clearance of BUN and creatinine was not greatly affected.

### 3.2. BMSC-CM Partially Preserves Histological Integrity of the Kidney in Rats Treated with Cisplatin

**Figure 3:**
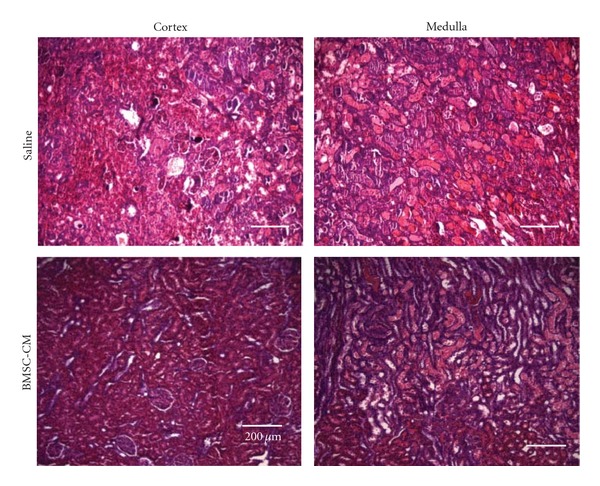
BMSC-CM protects the native architecture of the kidney during AKI. Hematoxylin and eosin staining of sections of kidneys from rats administered a lethal dose of cisplatin (7.5 mg/kg) and sacrificed at 5 days following cisplatin administration. The rats were treated at 3, 9, 24, 48, and 72 hours following cisplatin administration, with 1 mL of either physiological saline (*n* = 4), or BMSC-conditioned medium (*n* = 3) i.v. Scale bar = 200 *μ*m.

 From the results of the renal function markers, BUN and creatinine, it seemed likely that the BMSC-CM treatment was protecting the architecture of the kidney, thereby preserving GFR. When the rats were subjected to a lethal dose of cisplatin and then treated with saline (*n* = 4), H&E staining of the kidneys revealed cortical damage in the form of collapsed Bowman's capsule and proximal tubular necrosis, and outer medullary damage in the form of extensive tubular necrosis, deposition of debris and tubule casts, all consistent with cisplatin-mediated AKI (Figure 3). The injury was so severe that upon excision, the kidneys were grossly hemorrhagic and the normal tissue firmness was replaced with softness. The kidneys of the rats administered BMSC-CM (*n* = 3), on the other hand, sustained significantly less injury. H&E analysis revealed less tubular necrosis, diminished loss of brush borders, decreased denuding of tubular epithelium, and a reduction in apparent casts or accumulated debris. 

**Figure 4:**
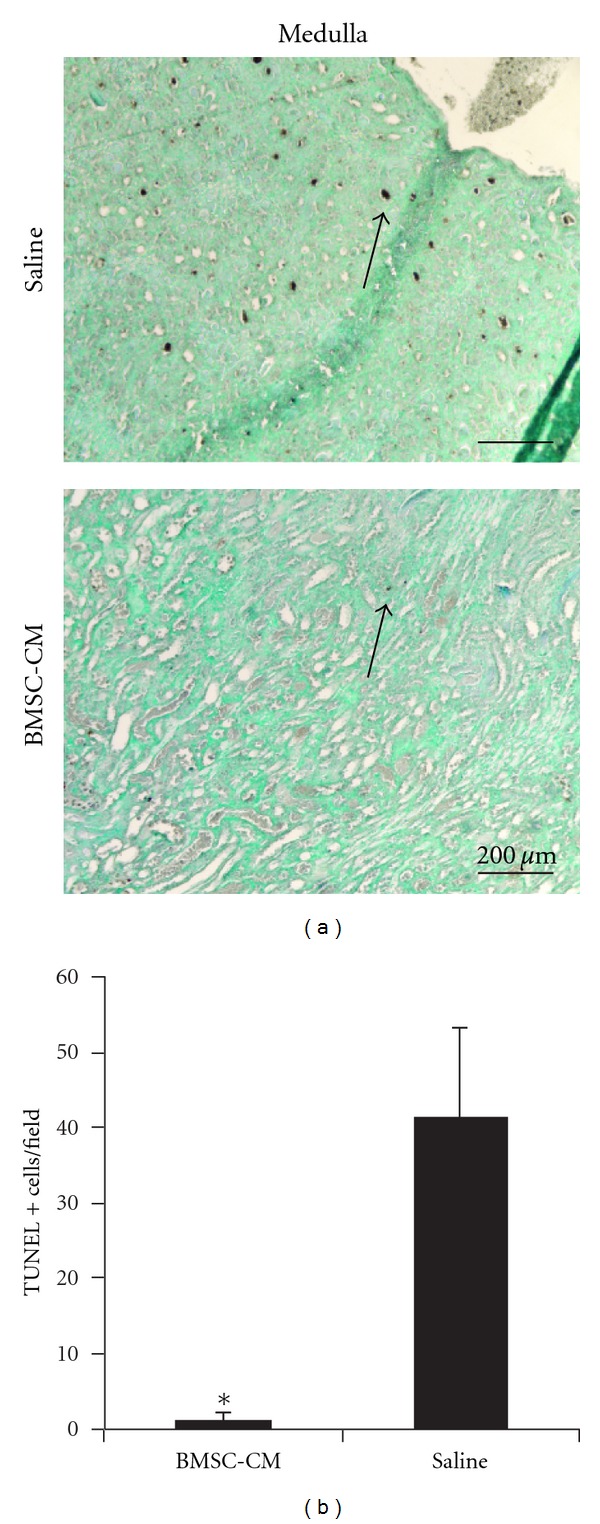
*BMSC-CM prevents apoptosis in the outer medulla.* (a) TUNEL staining was performed on sections of formalin fixed and paraffin-embedded kidneys from rats administered a lethal dose of cisplatin (7.5 mg/kg) and sacrificed at 5 days following cisplatin administration. The arrows indicate TUNEL positive cells. The rats were treated at 3, 9, 24, 48 and 72 hours following cisplatin administration, with 1 mL of either physiological saline (*n* = 4), or BMSC conditioned medium (*n* = 3) i.v. (b) Quantification of the TUNEL positive cells per field of view. Scale bar = 200 *μ*m. *P < 0.01 by Student's *T*-test.

We next sought to analyze apoptosis in the kidneys during cisplatin toxicity. TUNEL analysis on day-5 kidney sections revealed extensive apoptotic foci in the medulla of the vehicle-treated kidneys, consistent with injury patterns in the pars recta of the proximal tubule that are precipitated by cisplatin [33] (*n* = 4, Figure 4(a)). We did not observe significant TUNEL positive staining in the cortex of these animals. The observed medullary apoptosis in the vehicle-treated animals was not present in the BMSC-CM treated animals (*n* = 3). Periodic apoptotic cells were observed in the BMSC-CM treated kidneys, but there were approximately 10X more in the vehicle-treated kidneys, indicating far fewer apoptotic events in BMSC-CM-treated kidneys (Figure 4(b)).

**Figure 5:**
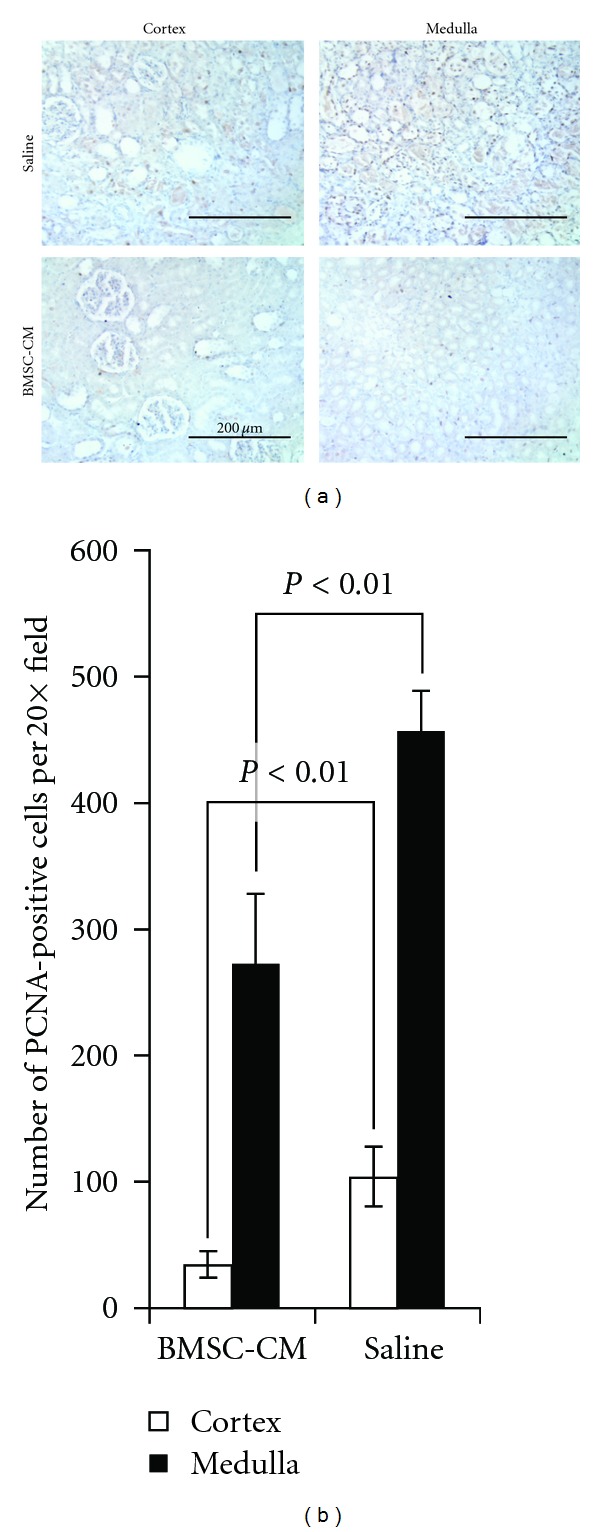
*Vehicle treated have a greater number of PCNA+ cells 5 days after cisplatin administration.* (a) PCNA staining was performed on sections of formalin fixed and paraffin-embedded kidneys from rats administered a lethal dose of cisplatin (7.5 mg/kg) and sacrificed at 5 days following cisplatin administration. The rats were treated at 3, 9, 24, 48 and 72 hours following cisplatin administration, with 1 mL of either physiological saline (*n* = 4), or BMSC conditioned medium (*n* = 3) i.v. (b) Quantification of the PCNA positive cells per field of view. Scale bar = 200 *μ*m.

 We then looked into the prevalence of markers of regeneration in the kidneys to test the hypothesis that BMSC-CM treatment may facilitate regeneration, which could contribute recovery of the animals. PCNA expression was far more extensive in the BMSC-CM treated kidneys (*n* = 3) than healthy control kidneys (*n* = 3) that only showed scarce PCNA-positive cells (less than one per 10X field on average, data not shown) (Figure 5) [34]. We quantified the number of PCNA positive cells in each 20X field, and found that the extent of positive staining in vehicle-treated kidneys was significantly higher than in BMSC-CM treated kidneys.

### 3.3. IL-10 Is Important for BMSC-CM Therapy in AKI

**Figure 6:**
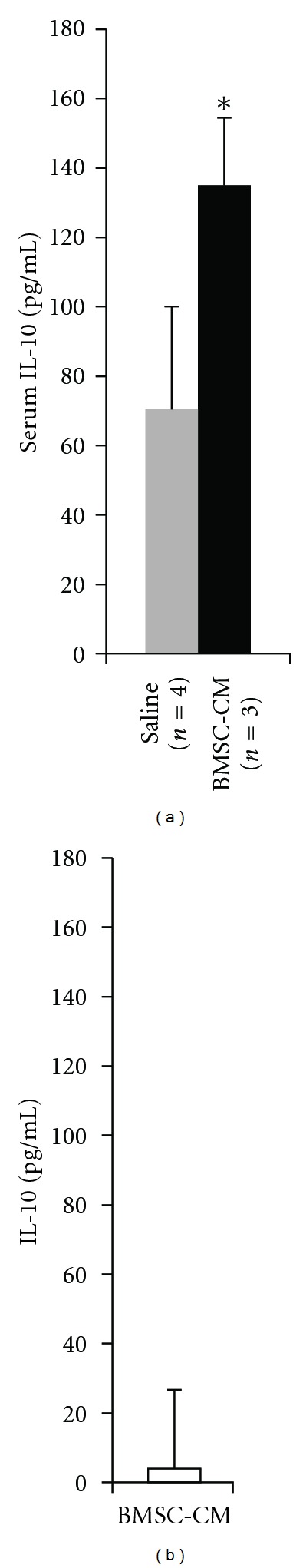
BMSC-CM significantly upregulates IL-10 in AKI rats, and BMSC-CM is not an exogenous source of IL-10. (a) Rat serum IL-10 was measured via ELISA from rats administered a lethal dose of cisplatin (7.5 mg/kg) and then treated with either vehicle or BMSC-CM at 3, 9, 24, 48, and 72 hours after cisplatin administration. Blood was collected via tail snip, and the serum was analyzed on day 3. *P < 0.05 by Student's *t*-test. (b) IL-10 was measured via ELISA in whole BMSC-CM.

 Since we saw such a strong response in the rats to BMSC-CM in AKI, we explored mechanistic implications of BMSC-CM therapy, in particular how BMSC-CM might influence the immune state of the animal during AKI. Our previous work revealed that during liver failure, the inflammatory state of rats treated with BMSC-CM was reduced compared to vehicle controls [24]: the vehicle-treated rats developed high systemic levels of IL-1*β*, TNF-*α*, IL-6, and IL-1ra, and low levels of IL-10, while BMSC-CM treated rats developed high levels of IL-10 and lower levels of IL-1*β*, TNF-*α*, IL-6 and IL-1ra. In other models as well, IL-10 upregulation has been shown to be a hallmark of BMSC therapy [13, 35], and has been shown to influence the expression of IL-1*β*, TNF-*α*, and IL-6 *in vitro* and *in vivo* [16, 36]. We evaluated whether BMSC-CM treatment resulted in enhanced IL-10 expression. The serum level of IL-10 in BMSC-CM treated rats (*n* = 3) three days after cisplatin administration was significantly higher than that in vehicle controls (*n* = 4, Figure 6(a)). To confirm that the IL-10 we measured did not arise from the BMSC-CM that we injected, we determined the amount of IL-10 in BMSC-CM. Even though the BMSC-CM was from human cells and the IL-10 we measured in the animals was rat, we nevertheless wanted to confirm there was no cross-reactivity in the ELISA and measured the BMSC-CM independently. We found that the minute positive reactivity of BMSC-CM in the ELISA could not account for the IL-10 seen in the serum (Figure 6(b)).

**Figure 7:**
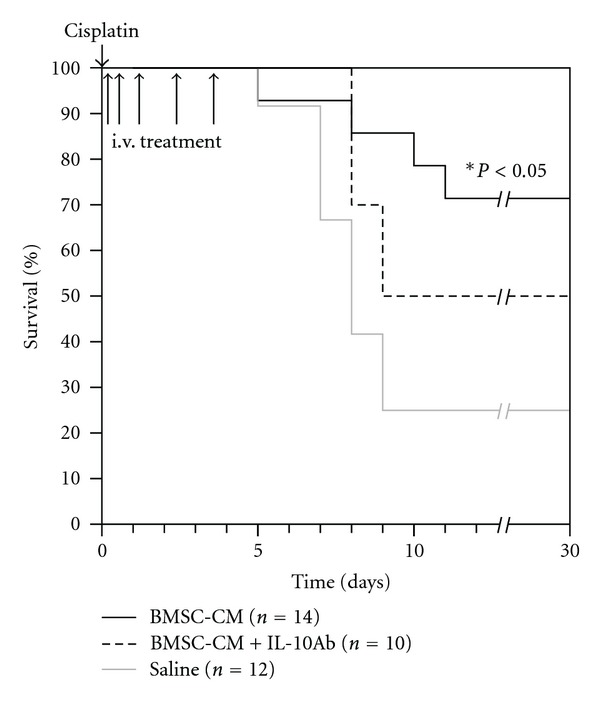
IL-10 antibody abates the survival benefit of BMSC-CM-administered to rats undergoing lethal cisplatin AKI. At hour 0, cisplatin dissolved in physiological saline (0.75 mg/mL) was administered i.p. to male, SAS-SD rats at a lethal dose (7.5 mg/kg). At hours 3, 9, 24, 48, and 72, 1 mL of either physiological saline (*n* = 12), BMSC-conditioned medium (*n* = 14) or BMSC-conditioned medium containing 4 *μ*g/mL neutralizing anti-rat IL-10 antibody (*n* = 10) were administered i.v. to the cisplatin AKI rats. They were monitored for survival for 30 days. *P < 0.05 for BMSC-CM compared to saline by Logrank test.

**Figure 8:**
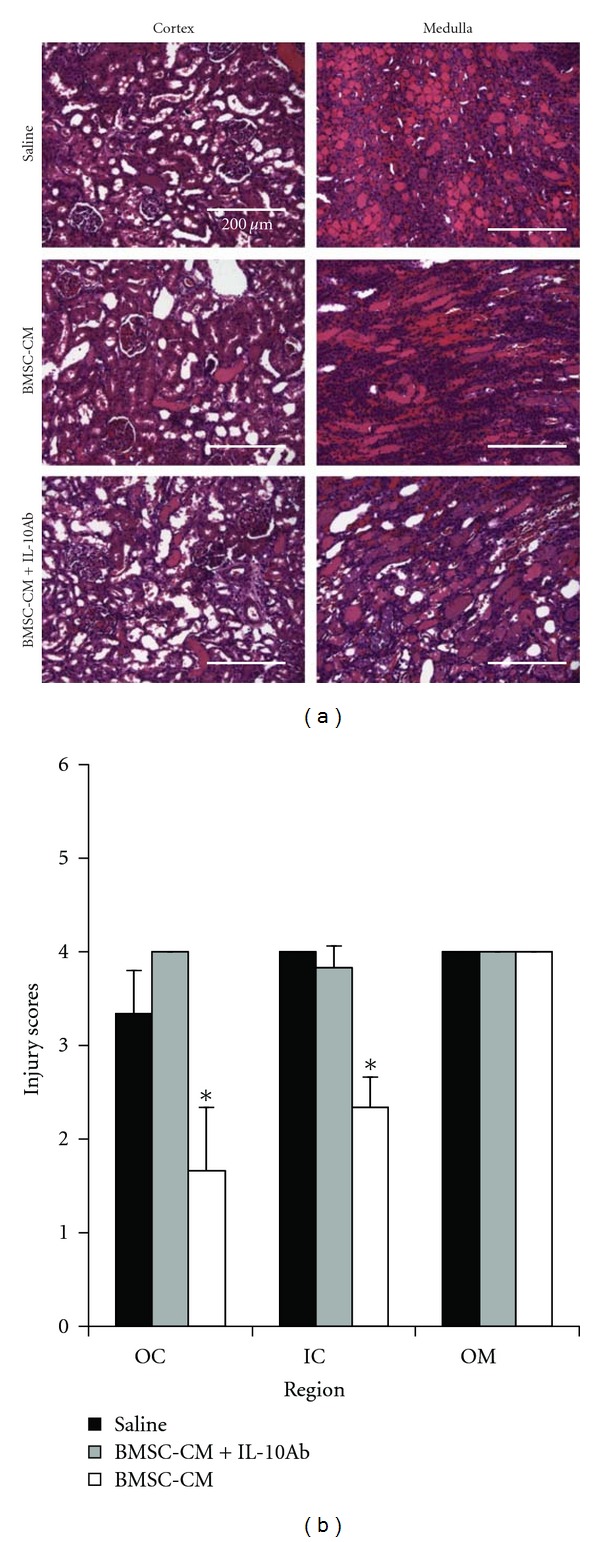
IL-10 antibody reduces the tissue sparing capacity of BMSC-CM. (a) H&E histological analysis of kidneys from cisplatin-treated rats administered either BMSC-CM plus a neutralizing IL-10 antibody (*n* = 3), BMSC-CM (*n* = 3), or saline (*n* = 3). Animals were sacrificed at 5 days after cisplatin for tissue harvesting. (b) Quantification of tissue injury in the outer cortex (OC), inner cortex (IC), and outer stripe of the outer medulla (OSOM) for rats treated with either BMSC-CM plus a neutralizing IL-10 antibody, BMSC-CM, or saline. Scale bar = 200 *μ*m. *P < 0.05 by Student's *t*-test.

**Figure 9:**
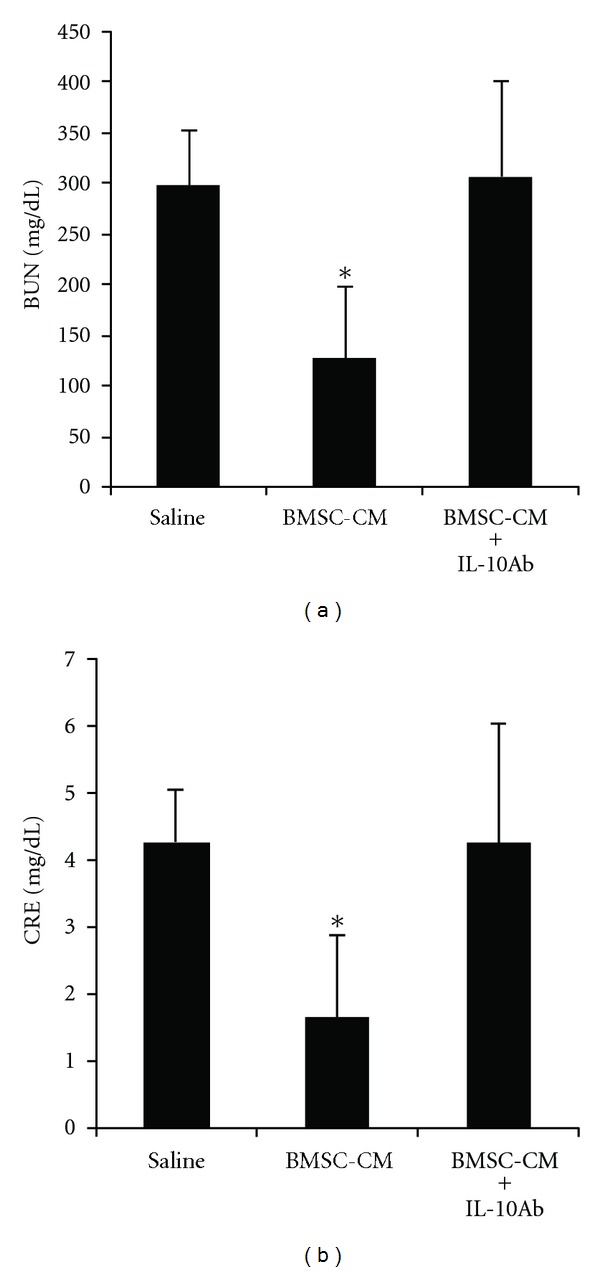
Protection of kidney function by BMSC-CM during AKI is abated with IL-10 antibodies. (a) Serum BUN and (b) serum creatinine values for rats subjected to cisplatin AKI and subsequently treated with either saline, BMSC-CM, or BMSC-CM with IL-10 neutralizing antibodies. Animals were sacrificed at 5 days after induction of AKI for blood collection. *P < 0.05 by Student's *t*-test.

**Figure 10:**
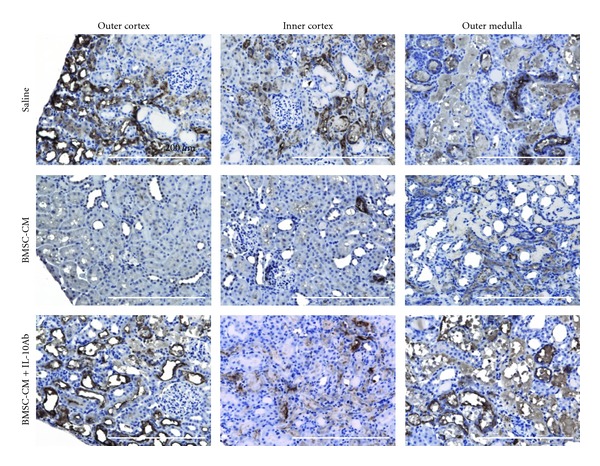
Kidney injury molecule-1 (KIM-1) positive staining is more extensive when IL-10 antibodies are coadministered with BMSC-CM compared to BMSC-CM alone. Immunohistochemical staining for KIM-1 of kidneys from AKI rats treated with either BMSC-CM plus a neutralizing IL-10 antibody (*n* = 3), BMSC-CM (*n* = 3), or saline (*n* = 3). Animals were sacrificed at 5 days after induction of AKI for tissue harvesting. Scale bar = 200 *μ*m.

Finally, to test the extent to which IL-10 may be responsible for the therapeutic effectiveness of the BMSC-CM treatment in AKI, we used the same survival model of cisplatin AKI (7.5 mg/kg) and coadministered BMSC-CM with neutralizing IL-10 antibody at 4 *μ*g per dose.  Figure 7 shows the diminished effect of BMSC-CM on the survival of animals when coadministered with neutralizing IL-10 antibody (*n* = 10) compared to the survival data presented in Figure 1: fewer animals survived when IL-10 antibody was coadministered with BMSC-CM. It should be noted that when tested for significance using the log rank test, the *P* value of the differences between BMSC-CM versus BMSC-CM with IL-10 antibody were 0.28, suggesting trends rather than significant increases or decreases in survival. Therefore, to further characterize the effect of IL-10 antibody on BMSC-CM administration, H&E histology was performed for the three treatment groups: saline (*n* = 3), BMSC-CM, (*n* = 3), or BMSC-CM with 4 *μ*g of IL-10 antibody (*n* = 3). Staining for the kidney injury marker, KIM-1 and serum analysis of kidney injury markers BUN and creatinine were also performed on the same groups. BUN and creatinine were chosen as common markers of kidney injury, and KIM-1 was chosen as a more specific and sensitive marker of direct kidney injury [27, 37, 38]. H&E highlighted differences between the severity of tissue injury as a result of IL-10 neutralization (Figure 8(a)) and histological scoring revealed a distinction between the kidneys treated with BMSC-CM and those with IL-10 neutralizing antibody (Figure 8(b)). In all groups, the injury to the outer medulla was too severe to see a difference between the treatment arms; however, in the inner and outer cortex, BMSC-CM treated animals exhibited significantly less injury than vehicle-treated and BMSC-CM plus neutralizing IL-10 antibody treated. Supportive of the survival results, the injury evident from H&E staining among the antibody treated animals was as severe as those treated with vehicle. As these results are partially subjective, future studies that quantify the injury more completely might further elucidate the effect of IL-10 neutralization on tissue injury. Nevertheless, these same results were reflected in BUN and creatinine levels (Figure 9) and KIM-1 staining (Figure 10). BMSC-CM treated animals exhibited significantly less BUN and creatinine accumulation and minimal KIM-1-positive staining compared to the marked rise of BUN and creatinine and extensive positive KIM-1 staining of the kidney in vehicle treated and BMSC-CM plus IL-10 antibodies treated groups. Clearly these results support the hypothesis that IL-10 is an important mediator of BMSC-CM therapy and suggest that through IL-10 upregulation, BMSC-CM protects the kidney from injury during AKI.

## 4. Discussion

We have shown that BMSC-CM alone can provide substantial support in AKI. Several studies have shown that transplanted BMSCs can provide survival benefit in AKI akin to what we observed. In one study, i.v. administration of human BMSCs (2 × 10^7^ cells/kg) in NOD/SCID mice at 24 hours following lethal cisplatin administration enhanced survival from 0% to 40% [39]. In another, i.p. administration (2 × 10^8^ cells/kg) was demonstrated to be sufficient for providing survival benefit to NOD/SCID mice, enhancing survival from 10% to 47% [40]. In our study, we used the secreted factors from the equivalent of 3 × 10^7^ cells/kg, which is within the same order-of-magnitude as other work demonstrating i.v. administration of BMSCs, which is why we might expect a similar result in terms of protection during AKI. Nevertheless, this is potentially not a direct comparison as the BMSC-CM consists of secreted factors from only 24 hours of conditioning, compared to permanent engraftment of transplanted BMSCs and continuous secretion of factors over time from the engrafted cells [41]. It should be recognized, however, that in most cases studied, cell survival is very transient.

Our results are consistent with other studies of BMSC-CM in AKI and show for the first time that human BMSC-CM can provide a survival benefit in the context of AKI. Bi and colleagues showed that in addition to BMSC transplantation conferring a survival benefit to mice undergoing cisplatin AKI, mouse BMSC-CM also provided a 60% increase in survival [42]. We observed a marked improvement in survival, which we hypothesize could be attributed to species differences of the CM [11]. However, since the study of Bi et al. was executed differently than ours with a different rodent species, mode of administration of BMSC-CM, treatment regimen, and BMSC-CM harvesting method, it is difficult to compare the results of the studies directly. Also, significant protection against kidney injury has been reported from administration of the ultracentrifuged fraction of BMSC-CM in mice undergoing rhabdomyolytic AKI [18]. The authors present evidence that microvesicles in the ultracentrifuged fraction provide benefit by horizontal gene transfer that is diminished with the coadministration of RNAse. Protection was, evidenced by histology, BUN/creatinine, and electron microscopy. Nevertheless, the authors did not explore whether the ultracentrifuged fraction provides a survival benefit, nor did they report whether the whole conditioned medium can provide a similar or superior benefit. In all, our results here have demonstrated the largest improvement of survival thus far of any BMSC study to our knowledge, and show that the secreted factors alone are sufficient for AKI resolution.

Our previous work has shown that the secreted factors from BMSCs, either administered in the form of conditioned medium (CM) or delivered continuously from an extracorporeal bioreactor, are capable of providing survival benefit to animals undergoing fulminant hepatic failure [22, 24]. We observed reversal of the proinflammatory cytokine profile in these animals, with a marked increase in the anti-inflammatory cytokine IL-10. IL-10 has been implicated in BMSC therapy previously in sepsis [13] and has been cited as one of the more influential factors upregulated during BMSC-mediated T-cell suppression [9, 43]. In sepsis, BMSCs were shown to upregulate IL-10 in macrophages via prostaglandin E2-mediated reprogramming. Others groups have shown that certain factors secreted by BMSCs can directly lead to IL-10 production [12], including TGF-*β* [44] and IL-6 [45], two proteins that are highly expressed by BMSCs [46, 47]. Clearly there are mechanistic differences that distinguish liver failure and sepsis from AKI, and it was not obvious that the same pattern of cytokine expression would be seen in AKI rats treated with BMSC-CM. Nevertheless, IL-10 has also been shown to be effective as a therapeutic when exogenously delivered to rodents undergoing AKI [48], confirming the important role that IL-10 can play in mitigating the disease. It comes as no surprise, therefore, that we observed upregulation of IL-10, and found that blocking the cytokine caused a decrease in the survival of animals treated with BMSC-CM.

 In an effort to leverage what we have learned about BMSC-CM and begin to translate it to clinical use, we have recently been working to scale up the extracorporeal bioreactor we developed to treat fulminant hepatic failure in rodents [22] to accommodate human use during dialysis for AKI [49]. In our studies of hepatic failure, we discovered that continuous delivery of MSC-secreted factors from a bioreactor can provide a superior therapeutic benefit compared to bolus administration of BMSC-CM [22]. This platform might also be applied to existing dialysis circuits to allow for delivery of BMSC-secreted factors directly into the bloodstream of patients with AKI undergoing dialysis. When seeded in the bioreactor, we showed the BMSCs are capable of remaining viable and functional while retaining their undifferentiated phenotype under flow conditions mimetic of clinical operation [49], which suggests that scale-up of the prototype device from our liver failure studies will likely result in a similarly active device for clinical use. The results we report here further support the hypothesis that the secreted factors from BMSCs delivered into the bloodstream of animals suffering from AKI can fundamentally change the outcome of those with AKI.

 Future work will be required to fully elucidate the mechanistic details of the phenomena reported here and will be necessary for rigorous comparison with whole cell transplantation. Our experiments have focused on conditioned medium obtained by concentrating BMSC supernatants with a 3 kDa cutoff filter. Small molecule mediators such as prostaglandin-E2 that are likely important for BMSC therapeutic activity were physically excluded in these studies [13, 50]. These and other mediators secreted by BMSCs are likely important for attenuating inflammation as well as providing mitogenic and angiogenic support to restore kidney function, and future efforts to uncover their effects will be informative. We also chose to use human BMSCs to retain relevance to trials with human BMSCs. However, to avoid potential xenogenicity, it may also be informative to repeat these studies with CM from rat BMSCs.

In conclusion, we have demonstrated that BMSC-secreted factors, independent of cell transplantation, are capable of stimulating endogenous anti-inflammatory programs via IL-10, leading to a significant survival benefit to rats undergoing cisplatin-induced AKI. These results suggest that the primary mechanism of BMSC therapy is based on the secreted factors of the cells, which should aid in the optimization of proper therapeutic protocols for treatment of AKI patients with BMSCs. It also suggests that alternative approaches to cell transplantation might also prove successful for administering BMSC therapy, including administration of the conditioned medium as a direct injectable, or continuous delivery of these secreted factors from bioreactors into the bloodstream of AKI patients, thereby circumventing potential risks associated with transplantation [51, 52]. In all, these results present a promising and innovative new approach to treating AKI that leverages the natural strengths of the BMSC secretome.

## Abbreviations

BMSC: Bone marrow stromal cell
IL-10: Interleukin 10
CM: Conditioned medium
PBMC: Peripheral blood mononuclear cell
FB: Fibroblast
H&E: Hematoxylin and eosin.
